# Activation of Bacterial Histidine Kinases: Insights into the Kinetics of the *cis* Autophosphorylation Mechanism

**DOI:** 10.1128/mSphere.00111-18

**Published:** 2018-05-16

**Authors:** Gaurav D. Sankhe, Narendra M. Dixit, Deepak K. Saini

**Affiliations:** a Centre for Biosystems Science and Engineering, Indian Institute of Science, Bangalore, India; b Department of Chemical Engineering, Indian Institute of Science, Bangalore, India; c Department of Molecular Reproduction, Development and Genetics, Indian Institute of Science, Bangalore, India; University of Iowa

**Keywords:** *Mycobacterium tuberculosis*, two-component signaling, autophosphorylation, *cis* autophosphorylation, histidine kinase, mathematical modeling

## Abstract

Two-component signaling systems (TCSs) are central to bacterial adaptation. However, the mechanisms underlying the reactions involving TCS proteins and their reaction rates are largely undetermined. Here, we employed a combined experimental and theoretical approach to elucidate the kinetics of autophosphorylation of three histidine kinases (HKs) of Mycobacterium tuberculosis, *viz.*, MtrB, PrrB, and PhoR, all known to play a role in regulating its virulence. Using wild-type and mutant proteins, we performed dimerization assays, thermophoretic-affinity measurements, and competition-based phosphorylation assays to establish that for HK, MtrB autophosphorylation occurs in *cis*, similar to what has been proposed for the PhoR and PrrB HKs. Next, to determine the kinetics of *cis* autophosphorylation, we used a quantitative high-throughput assay and identified a two-step mechanism of HK activation, involving (i) the reversible association of HK with ATP, followed by (ii) its phosphorylation. We developed a mathematical model based on this two-step *cis* mechanism that captured the experimental data. Best-fit parameter values yielded estimates of the extent of HK-ATP association and the rates of HK autophosphorylation, allowing quantification of the propensity of HK autophosphorylation. Our combined experimental and theoretical approach presents a facile, scalable tool to quantify reactions involving bacterial TCS proteins, useful in antibacterial drug development strategies.

**IMPORTANCE** Two-component systems consisting of an input-sensing histidine kinase (HK) and an output-generating response regulator (RR) are one of the key apparatuses utilized by bacteria for adapting to the extracellular milieu. HK autophosphorylation is shown to occur primarily in *trans* (intermolecular) and more recently shown to occur in *cis* (intramolecular). Although the catalysis of HK activation remains universal, the reaction scheme for evaluation of the kinetic parameter differs between these designs and *cis* mode largely remains unexplored. We combined experimental and theoretical approach to unravel two-step mechanism of activation of three *cis* mode HKs of M. tuberculosis. The new mathematical model yields best-fit parameters to estimate the rates of HK-ATP association and HK autophosphorylation.

## Abstract (precis)

Two-component systems consisting of an input-sensing histidine kinase (HK) and an output-generating response regulator (RR) are one of the key apparatuses utilized by bacteria for adapting to the extracellular milieu. HK autophosphorylation is shown to occur primarily in *trans* (intermolecular) and more recently shown to occur in *cis* (intramolecular). Although the catalysis of HK activation remains universal, the reaction scheme for evaluation of the kinetic parameter differs between these designs and *cis* mode largely remains unexplored. We combined experimental and theoretical approach to unravel two-step mechanism of activation of three *cis* mode HKs of M. tuberculosis. The new mathematical model yields best-fit parameters to estimate the rates of HK-ATP association and HK autophosphorylation.

## INTRODUCTION

Two-component signaling systems (TCSs) are central to prokaryotes and lower eukaryotes for sensing and responding to changes in their extracellular environments. A typical TCS consists of two proteins, a stimulus-sensing histidine kinase (HK) and a change-triggering response regulator (RR). In a typical reaction scheme, the HK undergoes autophosphorylation at a conserved histidine following stimulation by a specific environmental cue. It then serves as a phosphodonor species and transfers the phosphoryl group to a specific partner RR protein on a conserved aspartate residue. The changes in the phosphorylation status of RR orchestrate adaptive responses to the environmental cue mostly by altering its DNA binding that lead to change in the expression of downstream genes (1–3). The TCSs of pathogenic bacteria form attractive drug targets, as the survival of pathogenic bacteria under pressure from host immune responses has been shown to be adversely affected if the functionality of one or more of the TCSs is genetically compromised (4, 5). Because the stimuli triggering HKs and the genes modulated by RRs are often unknown (6), studies aim to develop drugs for blocking the autophosphorylation of HKs or the phosphotransfer from HKs to their cognate RRs, which are more general and conserved reactions (7). Accurate biochemical characterization of autophosphorylation and phosphotransfer reactions is therefore likely to be useful in accelerating antibacterial drug development.

Most bacteria contain many distinct TCS proteins, for instance, Mycobacterium tuberculosis contains about 30 TCS proteins, Escherichia coli about 62, and Myxococcus xanthus about 270 (8). In addition to cognate HK→RR phosphotransfer, in some bacteria such as M. tuberculosis, HKs can “cross phosphorylate” or “cross talk” with noncognate RRs, increasing the number of reactions involving the TCS proteins (9). However, even in the best-studied systems like E. coli, the autophosphorylation kinetics of only 8 of the 31 HKs have been investigated (2, 10–14). In addition, effective biochemical characterization must define an appropriate reaction scheme for autophosphorylation, which also remains to be investigated for most HKs. Autophosphorylation can happen in *cis* (intramolecular) or in *trans* (intermolecular) (3, 15). While autophosphorylation in *trans* was historically reported (16–18), evidence of *cis* mode are reported more recently (19, 20). The kinetics of autophosphorylation occurring in *trans*, involving the unphosphorylated protein kinase and ATP as the substrates and the phosphorylated protein kinase as enzyme, has been theoretically analyzed (21) and described using the classical Michaelis-Menten formalism, which may not be optimal on account of the enzyme and product being the same molecule. The assumption of such formalisms that (i) a negligible amount of the product is formed and (ii) excess enzyme is present while evaluating the initial velocities may be disproportionate in HK-limiting conditions owing to low *in vitro* HK activity reported previously (22–24). Also this formulation would not be applicable in *cis* mode, as there the enzyme converts itself into the product. In *M*. *tuberculosis*, recent structural studies suggest *cis* autophosphorylation for the HK PhoR (25). *cis* autophosphorylation has been argued for the HK PrrB as well: the length and handedness of the loop joining the α1 and α2 helices in the dimerization and histidine phosphorylation (DHp) domain of PrrB are similar to HKs autophosphorylating in *cis* in E. coli, such as PhoR and ArcB (26, 27). Beyond TCSs, a wide spectrum of protein kinases have also been argued to be autophosphorylated in *cis* (28). However, a kinetic scheme for analyzing autophosphorylation in *cis*, handling the aforementioned constraints, is lacking. Our goal in the present study was to develop such a scheme.

We first establish a scheme for elucidating the mechanism of autophosphorylation of the HKs, using MtrB of *M*. *tuberculosis* as a model using a variety of techniques and show that it occurs through a *cis* mechanism. We also report an affinity measurement scheme to establish that our findings are not limited by conformational artifacts associated with mutated proteins, which are necessary for elucidating *cis*/*trans* mechanisms. We next utilize a high-throughput assay (HTA) to measure the reaction kinetics using MtrB and extend it to two other HKs, PrrB and PhoR, of *M*. *tuberculosis*, argued previously to also autophosphorylate in *cis*. We then develop a mathematical model to analyze the data and determine the rates of autophosphorylation for them. The rate measurements together with the model unravels a two-step *cis* autophosphorylation mechanism and allows quantification of the propensity of this reaction across HKs. Our study provides a facile, quantitative template for investigating reactions involving bacterial TCS proteins in a high-throughput manner, which may aid the identification of drug targets (29) and provide insights into the design principles governing TCS signaling and the survival and adaptive responses in bacteria (30–32).

## RESULTS

For determination of the autophosphorylation rates of HKs, we first had to establish the experimental conditions where maximal HK autophosphorylation is recorded and then identify the mechanism of their autophosphorylation, i.e., whether it occurs in *cis* or *trans*. This was essential, as the reaction steps vary depending on the mechanism and dictate the nature of the kinetic measurements. For our study, we decided to use three functionally characterized HKs of *M. tuberculosis*, *viz.*, MtrB, PrrB, and PhoR (31), which have been proposed to play a role in regulating *M. tuberculosis* pathogenicity (33).

### Optimal HK autophosphorylation occurs in the presence of Mg^2+^ ions.

Given that divalent cations are necessary for HK autophosphorylation (34), we first tested for the preferred divalent cation in the autophosphorylation reaction for each of the selected HKs before kinetic measurements. Autophosphorylation was performed in the presence of 10 mM Ca^2+^, Mg^2+^, or Mn^2+^ ions (all chloride salts) for 90 min. Although the presence of Ca^2+^ and Mn^2+^ did allow autophosphorylation to occur, Mg^2+^ ions were the most preferred as they facilitated maximal autophosphorylation (see Fig. S1 in the supplemental material). No autophosphorylation was seen in the absence of cations. We therefore used Mg^2+^ for all further studies.

10.1128/mSphere.00111-18.1 FIG S1  Effects of divalent cations on HK autophosphorylation. Autophosphorylation reactions were performed in the presence of 10 mM Ca^2+^, Mg^2+^, or Mn^2+^ ions for 90 min per the protocol described in Materials and Methods. (A) MtrB, (B) PrrB, and (C) PhoR. The lane marked negative control (NC) does not contains divalent cation. In each panel, the autoradiogram (top) and the corresponding Coomassie brilliant blue (CBB)-stained gel (bottom) are shown. The graph above the autoradiogram represents the densitometric analysis of the bands in the autoradiogram. The data in graph are shown as means ± SD (*n* = 3). Download FIG S1, TIF file, 1.0 MB. Copyright © 2018 Sankhe et al. 2018 Sankhe et al. This content is distributed under the terms of the Creative Commons Attribution 4.0 International license.

### MtrB autophosphorylation occurs in *cis* (intramolecular).

**FIG 1:**
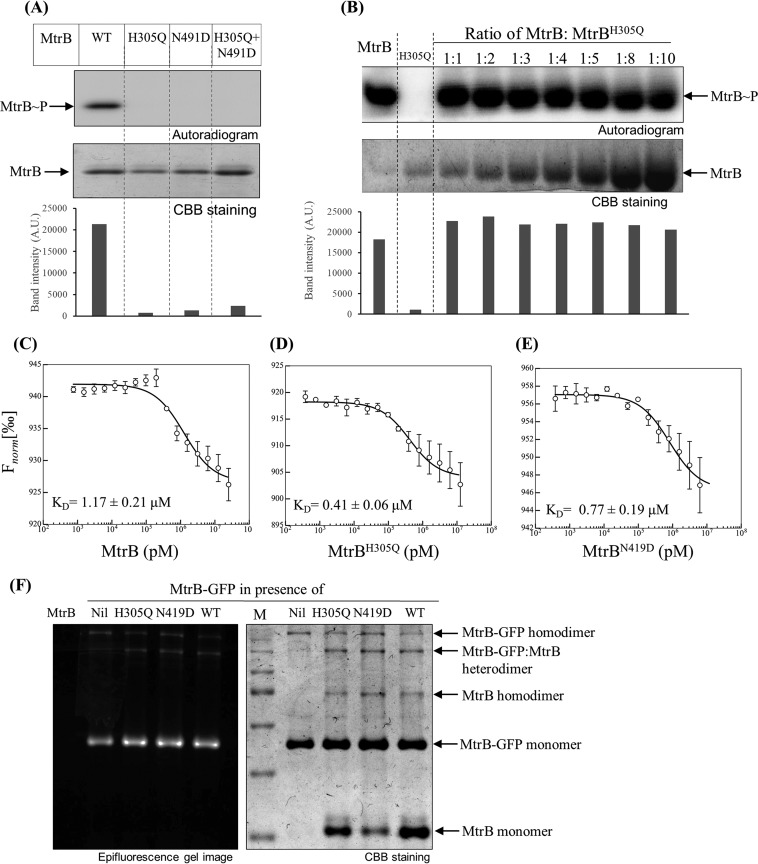
Analysis of HK autophosphorylation using mutant complementation assay. (A) Autophosphorylation analysis of MtrB proteins. Wild-type (WT) MtrB or the MtrB^H305Q^ or MtrB^N419D^ mutant was tested in autophosphorylation reaction for 90 min per the protocol in Materials and Methods. The graph below the autoradiogram represents densitometric analysis of the autoradiogram (in arbitrary units [A.U.]). (B) Analysis of *trans* phosphorylation by competition experiment. MtrB and MtrB^H305Q^ proteins were coincubated in various molar ratios as indicated for 10 min. The autophosphorylation reaction was performed for 60 min. (Top) Autoradiogram; (bottom) CBB-stained gel. The graph below the autoradiogram represents densitometric analysis of the autoradiogram. (C to E) Microscale thermophoresis measurements for determination of interaction affinities of MtrB-GFP with WT MtrB (C), MtrB^H305Q^ (D), and MtrB^N419D^ (E). All graphs were best fit to means ± standard errors of the means (SEM) from three independent experiments. *F*_norm_, normal fluorescence. (F) MtrB dimerization analysis using nonreducing SDS-PAGE and fluorescence imaging assay by coincubating 2 µM MtrB-GFP with MtrB, MtrB^H305Q^, or MtrB^N419D^ (at a concentration of 5 µM). Imaging was done per the protocol described in Materials and Methods. Lane M contains molecular size standards.

As described above, the HKs can undergo phosphorylation in either a *cis* (intramolecular) or *trans* (intermolecular) mechanism. PrrB and PhoR have already been proposed to undergo phosphorylation through a *cis* mode (25, 26). We thus used the HK MtrB to identify its phosphorylation mechanism. For this, we first generated a phosphorylation site (His) mutant (in the dimerization and histidine phosphorylation DHp domain [35]), MtrB^H305Q^, and another mutant defective in ATP binding (in the catalytic ATPase domain), MtrB^N419D^, both of which by themselves were phosphorylation defective (Fig. 1A). These mutant proteins were coincubated to record the occurrence of *trans* phosphorylation, if any. The rationale of this experiment is that in a *trans* model, phosphorylation of the ATPase mutant MtrB^N419D^ can occur as it has a viable phosphoacceptor His site by dimerization with a His site mutant, which is capable of binding ATP. However, we found no phosphorylated MtrB species in this coincubation assay (Fig. 1A). This conspicuous absence of the phosphorylation signal suggested that the mutated proteins are unable to complement, indicative of a *cis* autophosphorylation mechanism. In this model, the ATP bound to the catalytic domain (ATPase_C) phosphorylates His in the DHp domain of the same molecule; hence, a defect in either the ATP domain or His site will render them phosphorylation defective.

To validate this, we performed a competition experiment by adding increasing amounts of MtrB^H305Q^ in the autophosphorylation reaction of the green fluorescent protein (GFP)-tagged wild-type MtrB protein, as reported in previous studies where the *cis*/*trans* mechanism was probed (19, 27). In this assay, if *trans* autophosphorylation is a viable mechanism, the MtrB^H305Q^ mutant should compete with the wild-type MtrB and will show a reduction in the phosphorylation of the wild-type MtrB protein. Here also we failed to record any reduction in MtrB autophosphorylation, which competed with equimolar concentrations and up to 10-fold concentrations of the mutated protein, reinforcing the *cis* autophosphorylation mechanism (Fig. 1B).

Given that histidine kinases are known to form stable homodimers (19), the above observations could also result due to lack of heterodimer formation in the complementation or competition assay on account of preformed homodimers in the proteins. To address this, we performed microscale thermophoresis studies, wherein rates of association of various MtrB proteins (wild type and mutants) were determined using GFP-tagged MtrB (MtrB-GFP). We first validated that the MtrB-GFP protein is as active as untagged MtrB protein (Fig. S2A) and then subjected it to microscale thermophoresis (MST) analysis using variants of untagged protein for binding analysis. The dissociation constant (*K*_*D*_) obtained for wild-type MtrB was 1.17 ± 0.21 µM, while the *K*_*D*_s were 0.41 ± 0.06 µM for MtrB^H305Q^ and 0.77 ± 0.19 µM for MtrB^N419D^ (Fig. 1C, D, and E), all very close and slightly lower for mutant proteins. The dissociation constants suggest that the dimerization of MtrB-GFP with its wild-type protein and mutant MtrB^H305Q^ and MtrB^N419D^ proteins occurs at the same affinities and rules out gross structural changes in the mutants that could influence their ability to dimerize, similar to what has been shown by Ashenberg and coworkers using the fluorescence resonance energy transfer (FRET) competition assay (27). Of note, the apparent dissociation constants of dimerization obtained are in the range of that for the HK CheA (*K*_*D*_ = 0.4 µM) (11) known to autophosphorylate in *trans*, thus reinforcing the fact that the affinities of homodimers are in the range that allows heterodimer formation.

10.1128/mSphere.00111-18.2 FIG S2  (A) Validation of autophosphorylation activity of MtrB-GFP. Purified MtrB-GFP (2 µM) and MtrB (5 µM) were tested in autophosphorylation reaction for 90 min per the protocol described in Materials and Methods. The graph below represents the densitometric analysis of the autoradiogram. (B) CD spectroscopy analysis. CD spectra of MtrB, MtrB^H305Q^, and MtrB^N419D^. The mutant proteins show similar degrees of secondary structure as the wild-type protein, indicating that the mutations do not substantially perturb the gross folding of the protein. Download FIG S2, TIF file, 1.2 MB. Copyright © 2018 Sankhe et al. 2018 Sankhe et al. This content is distributed under the terms of the Creative Commons Attribution 4.0 International license.

To further confirm the ability of wild-type MtrB and its mutants to form heterodimers, the wild-type and mutant MtrB proteins were coincubated with the recombinant MtrB-GFP protein and were resolved on a nonreducing 15% SDS-polyacrylamide gel. Here we recorded the presence of all the monomeric and dimeric species as anticipated. Distinct monomers of untagged and GFP-tagged MtrB, homodimers of tagged and GFP-tagged MtrB, along with the heterodimer of GFP-MtrB with untagged MtrB in the Coomassie brilliant blue (CBB)-stained gel as well as in fluorescence imaging were observed, confirming that the wild-type and mutant MtrB proteins do heterodimerize (Fig. 1F). The mutant proteins were also subjected to circular dichroism (CD) spectroscopy analysis, where no gross difference in the secondary structures of MtrB and the mutant proteins MtrB^H305Q^ and MtrB^N419D^ were recorded (Fig. S2B). Overall, our analysis allowed us to establish that MtrB autophosphorylation occurs by a *cis* mechanism, and using it as the template, we next analyzed the kinetic parameters and developed the mathematical model.

### Equivalence of the HTA platform and PAGE/autoradiography.

**FIG 2:**
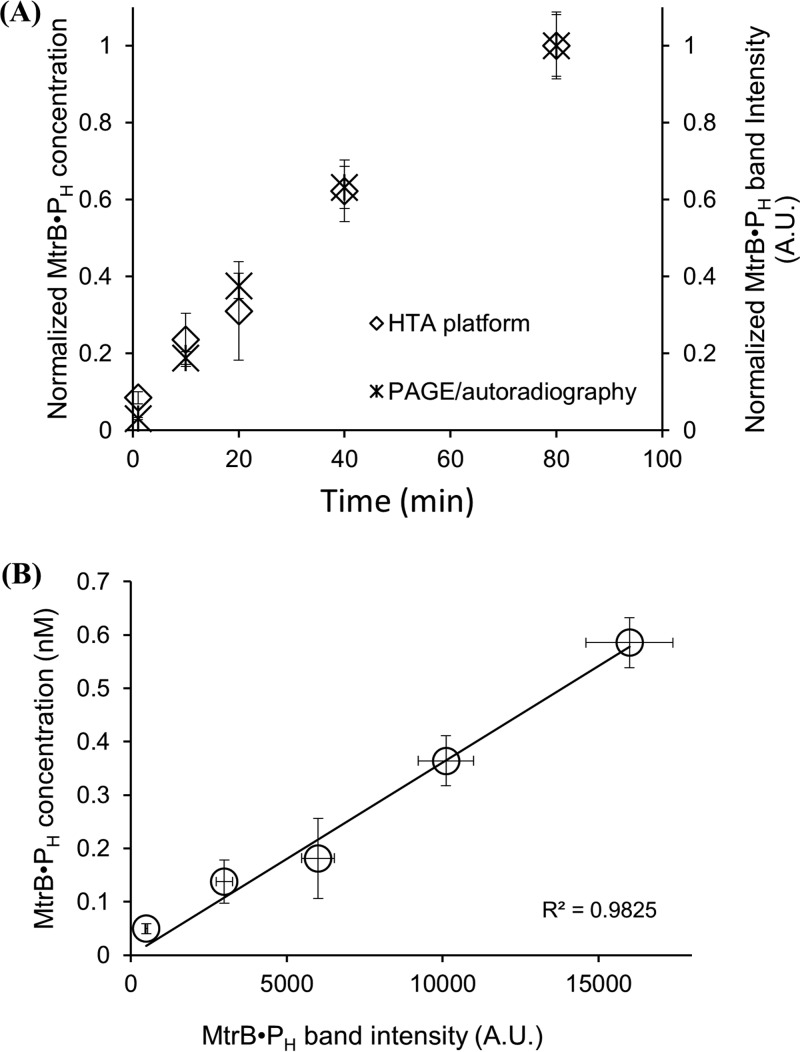
Equivalence of HTA platform and PAGE/autoradiography for determining HK autophosphorylation kinetics. (A) Evolution over time of labeled MtrB measured using HTA and PAGE/autoradiography independently and normalized to the maximum measured values in the respective assays. MtrB (5 µM) and 50 µM unlabeled ATP and 50 nM labeled ATP were used for the reaction per the protocol described in Materials and Methods. The left-hand *y* axis shows normalized MtrB·P_H_ concentration obtained using HTA, and the right-hand *y* axis shows normalized MtrB·P_H_ band intensities. The evolution over time of the normalized concentrations of MtrB·P_H_ measured using the HTA platform was nearly identical to the normalized intensities measured using PAGE/autoradiography (*P* = 0.8 using two-tailed Student’s *t* test with paired samples and unequal variance). (B) Band intensities from PAGE/autoradiography showed linear correlation with corresponding concentrations of labeled MtrB obtained using the HTA platform (see Fig. S3 in the supplemental material). The data are shown as means ± standard deviations (SD) (*n* = 3).

Given that autophosphorylation involves incorporation of the ^32^P-labeled phosphoryl moiety from ATP onto an HK molecule, technically any assessment platform wherein labeled HK can be separated from unincorporated ATP can be utilized, including techniques like electrophoresis, chromatography, or filtration (34, 36–38). Here, we employed a 96-well filter plate platform (referred to as HTA assay henceforth), which allows quantification of acid-labile phosphohistidine in a high-throughput manner. In this assay, the labeled HK is retained on the membrane and unincorporated ATP is washed off using cold washing buffer, which does not affect the phosphohistidine moiety, thereby facilitating quantification of HK phosphorylation. Existing evidence of TCS phosphorylation reactions and information on their kinetics predominantly comes from conventional autoradiography and SDS-PAGE-based assays (2, 10, 17). Although a 96-well nitrocellulose filter-based plate assay was utilized to study autophosphorylation of the HK CheA (39), its comparative analysis with standard PAGE/autoradiography assay was lacking. Therefore, we first tested the equivalence of the HTA platform by the conventional PAGE/autoradiography technique. For this, we performed autophosphorylation analysis of MtrB concomitantly on the HTA platform and the PAGE/autoradiography setup. The evolution over time of the normalized concentrations of labeled MtrB (denoted [HK·P_H_]; see below) measured using the HTA platform was nearly identical to the normalized intensities measured using PAGE/autoradiography (Fig. 2A; the corresponding gel and autoradiographs are shown in Fig. S3). Further, the band intensities obtained from autoradiography showed a linear correlation with [HK·P_H_] from the HTA platform, yielding a calibration curve of band intensity with respect to the concentration (Fig. 2B). This close agreement gave us confidence that the HTA platform could be used instead of the PAGE/autoradiography technique, which is an indirect technique. To characterize the kinetics of HK autophosphorylation, we utilized the HTA platform for all our subsequent assays.

10.1128/mSphere.00111-18.3 FIG S3  Analysis of MtrB autophosphorylation kinetics using PAGE/ autoradiography. (A) MtrB (5 µM) was phosphorylated for the indicated times and analyzed by SDS-PAGE followed by autoradiography (as per the data shown in Fig. 1). The autoradiograph (top) and the corresponding Coomassie brilliant blue (CBB)-stained gel (bottom) are shown. (B) The graph is a representation of the densitometric analysis of the bands in the autoradiogram. The data in the graph are shown as means ± SD (*n* = 3). Download FIG S3, TIF file, 0.6 MB. Copyright © 2018 Sankhe et al. 2018 Sankhe et al. This content is distributed under the terms of the Creative Commons Attribution 4.0 International license.

### HK autophosphorylation kinetics.

**FIG 3:**
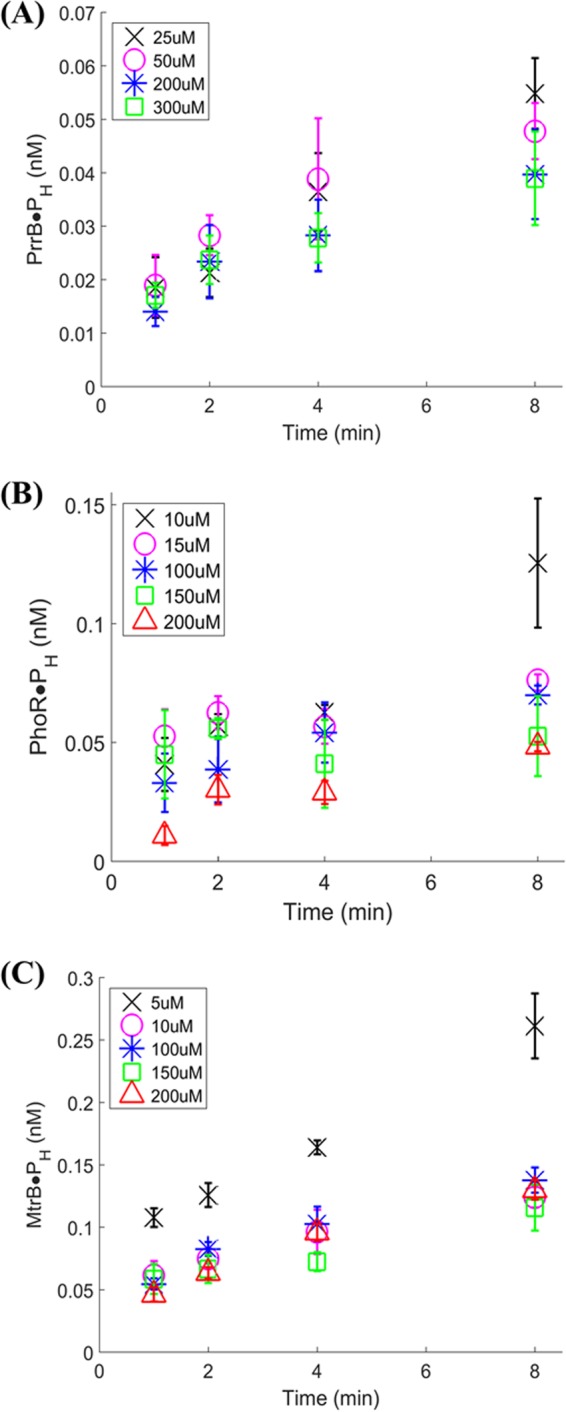
Time course analysis of formation of phosphorylated HK. Amount of labeled HK and PrrB (A), PhoR (B), and MtrB (C) formed at various time points determined using the HTA platform in the presence of various ratios of unlabeled/labeled ATP used in the reaction. The amounts of unlabeled ATP employed that were used along with ~50 nM labeled ATP are indicated. HKs were used at the following concentrations in the reaction, 10 µM for PrrB and PhoR and 5 µM for MtrB. The data are shown as means ± SD (*n* = 3).

We first measured the concentrations of labeled HK, denoted [HK·P_H_], which is equal to [HK~P_H_] + [HK-ATP_H_] (see mathematical model below), at different time points from the start of the experiment, for fixed initial HK and different initial ATP concentrations (Fig. 3). (Note that we use the subscript H throughout to denote labeled entities and subscript C for unlabeled entities.) To capture initial reaction velocities, we made frequent measurements until 8 min. For any ATP concentration, [HK·P_H_] rose sharply to a value much larger than zero at the earliest measured time point (1 min), indicating the rapid association between ATP and HK. Subsequently, [HK·P_H_] increased more gradually and nearly linearly with time. For instance, for PhoR, with [ATP_C_] at the start of the experiment (at time 0) ([ATP_C_]_0_) = 100 µM (unlabeled ATP) and [ATP_H_]_0_ = 55 nM (labeled ATP), [HK·P_H_] was ~0.03 nM at *t* = 1 min and then increased gradually to ~0.07 nM over the next 7 min (Fig. 3B). (Here, the concentrations were obtained by converting the dpm counts measured into nanomolar using the known specific activity of the labeled ATP as shown in calculations.) In other words, about 50% of the total association between PhoR and ATP, recorded after 8 min of incubation, occurred within the first 1 min of the reaction. For MtrB, about 50% of the association recorded at 8 min was seen within the first minute of incubation (when [ATP_C_]_0_ = 100 µM and [ATP_H_]_0_ = 55 nM). The corresponding value for PrrB is ~40% (with [ATP_C_]_0_ = 50 µM and [ATP_H_]_0_ = 55 nM). This suggested a two-step autophosphorylation process, where the HK and ATP associated rapidly to form complexes ([HK-ATP_C_] and [HK-ATP_H_]) in the first step, which yielded phosphorylated HK ([HK~P_C_] and [HK~P_H_]) in the second slower step.

The data present two additional insights into the two-step process. First, the separation of the time scales of these two steps, *viz*., binding and catalytic activation, along with the incomplete consumption of HK in the initial step despite the large excess of ATP suggested that the initial association is rapid and reversible. The formation of complexes may therefore be assumed to reach pseudoequilibrium instantaneously. Interestingly, second, the concentration of labeled HK, [HK·P_H_], declined as the amount of unlabeled ATP, [ATP_C_]_0_, was increased while keeping the amount of labeled ATP, [ATP_H_]_0_, constant. For instance, with [ATP_C_]_0_ = 200 µM, [HK·P_H_] was ~0.01 nM at *t* = 1 min and ~0.05 nM at *t* = 8 min, lower than the values mentioned above with [ATP_C_]_0_ = 100 µM. This trend is expected when HK is limiting; the labeled and unlabeled ATP will compete for HK even at the initial time points. More ATP_C_ relative to ATP_H_ in such a scenario would lead to ATP_C_ outcompeting ATP_H_ in associating with HK, and hence, lower values of [HK·P_H_] are obtained. If HK was not limiting, the concentration of labeled HK, [HK·P_H_], would not change with changes in unlabeled ATP concentrations. We found similar trends with the two other HKs, PrrB (Fig. 3A) and MtrB (Fig. 3C). The concentrations of HK employed were generally comparable to the total ATP levels employed. The limitation in HK could thus be attributed to a fraction of the employed HK being capable of ATP binding and autophosphorylation. We employed these insights in constructing a mathematical model of the kinetics of *cis* HK autophosphorylation presented below.

### Mathematical model.

We envision the following reactions following the exposure of HK to ATP:

(1) $$\begin{array}{c} HK+ATP_{H}\overset{K_{e}}{⇌}HK\text{-}ATP_{H}\\ \begin{array}{c} \begin{array}{c} HK\text{-}ATP_{H}\overset{k_{p}}{\to }HK∼P_{H}+ADP\\ HK+ATP_{C}\overset{K_{e}}{⇌}HK\text{-}ATP_{C} \end{array}\\ HK\text{-}ATP_{C}\overset{k_{p}}{\to }HK∼P_{C}+ADP \end{array} \end{array}$$

where *K*_*e*_ is the equilibrium constant and *k_p_* is the phosphorylation rate.

Here, labeled ATP (denoted ATP_H_) and unlabeled ATP (denoted ATP_C_) rapidly form reversible complexes with the HK, *viz.*, HK-ATP_H_ and HK-ATP_C_, respectively. Then, ATP in the complexes phosphorylates HK, yielding the phosphorylated analogs, HK~P_H_ and HK~P_C_. Note that HK dimerization is unimportant, given that autophosphorylation occurs in *cis*. However, this is not the case when the phosphorylation occurs in *trans*, and dimerization becomes an essential step and has to be accounted for in determining rates. We neglect dephosphorylation of the HK~P species primarily considered to be ADP driven (14), as we focus on the early stages of the experiment. The evolution of the concentration of the phosphorylated species over time is then given by

(2) $$\begin{array}{c} \frac{d[HK\sim P_{H}]}{dt}=k_{p}[HK{\text{-ATP}}_{H}]=k_{p}k_{e}[HK{]\text{[ATP}}_{H}]\\ \frac{d[HK\sim P_{C}]}{dt}=k_{p}[HK{\text{-ATP}}_{C}]=k_{p}k_{e}[HK{]\text{[ATP}}_{C}] \end{array}$$

where we have assumed that the reversible complex formation in equation 1 is rapid and hence in pseudoequilibrium (see Results), so that

(3) $$\begin{array}{c} \left[HK{\text{-ATP}}_{H}]=K_{e}[HK]{\text{[ATP}}_{H}\right]\\ \left[HK{\text{-ATP}}_{C}]=K_{e}[HK]{\text{[ATP}}_{C}\right] \end{array}$$

Note that *K*_*e*_, the equilibrium constant, and *k*_*p*_, the phosphorylation rate, are identical for the labeled and unlabeled forms of ATP. Yet, we track the forms separately because the former form alone can be detected in measurements. To solve the above equations, we invoke the following species conservation relations:

(4) $$\begin{array}{c} {}[HK]=\phi {\left[HK\right]}_{0}-([HK{\text{-ATP}}_{H}]+[HK\sim P_{H}]+[HK{\text{-ATP}}_{H}]+[HK\sim P_{C}])\\ \begin{array}{c} \begin{array}{c} {}[{\text{ATP}}_{H}]={\left[{\text{ATP}}_{H}\right]}_{0}-(\left[HK{\text{-ATP}}_{H}]+[HK\sim P_{H}]\right) \end{array}\\ {}[{\text{ATP}}_{C}]={\left[{\text{ATP}}_{C}\right]}_{0}-(\left[HK{\text{-ATP}}_{C}]+[HK\sim P_{C}]\right) \end{array} \end{array}$$

Here, [HK]_0_ is the amount of HK at the start of the experiment (time *t* = 0) and ϕ is the fraction that is active and proficient in ATP binding and phosphorylation. Similarly, [ATP_H_]_0_ and [ATP_C_]_0_ are the initial concentrations of the two forms of ATP. We consider the case where ATP is in large abundance so that [ATP_H_] and [ATP_C_] remain approximately constant throughout the duration of recording and close to their initial values, [ATP_H_]_0_ and [ATP_C_]_0_, respectively. This assumption is consistent with HK being limiting. Using this approximation and equation 3 in the first equation of the set of equations shown for equation 4 and rearranging terms, it follows that

(5) $$\text{ [HK] = }\frac{\phi {\text{[HK]}}_{0}\;-{\text{ ([HK\textasciitilde{}P}}_{\text{H}}{\text{] + [HK\textasciitilde{}P}}_{\text{C}}\text{])}}{1+{\text{([ATP}}_{\text{H}}{\text{]}}_{0}\;{\text{+ [ATP}}_{\text{C}}{\text{]}}_{0}\text{)}K_{e}}$$

Substituting equation 5 in equation 2 and adding the two equations in equation 2 yields

(6) $$\frac{d{\text{([HK\textasciitilde{}P}}_{\text{H}}{\text{] + [HK\textasciitilde{}P}}_{\text{C}}\text{])}}{dt}\text{ = }A\text{(}\phi {\text{[HK]}}_{0}\;-{\text{([HK\textasciitilde{}P}}_{\text{H}}{\text{] + [HK\textasciitilde{}P}}_{\text{C}}\text{]}))$$

where $A=k_{p}K_{e}{\text{([ATP}}_{\text{H}}{\text{]}}_{0}{\text{+ [ATP}}_{\text{C}}{\text{]}}_{0}\text{)}/(1+K_{e}{\text{([ATP}}_{\text{H}}{\text{]}}_{0}{\text{+ [ATP}}_{\text{C}}{\text{]}}_{0}\text{))}$ is a constant. Solving equation 6 with the initial condition that [HK~P_H_] + [HK~P_C_] = 0 at time *t* = 0 yields

(7) $${\text{[HK\textasciitilde{}P}}_{\text{H}}\text{]}+{\text{[HK\textasciitilde{}P}}_{\text{C}}\text{]}=\phi {\text{[HK]}}_{0}\text{(1}-e^{-At}\text{)}$$

Substituting equation 7 in equation 5 yields the evolution of [HK] over time:

(8) $$\text{[HK] = }\frac{\phi {\text{[HK]}}_{0}e^{-At}}{1+{\text{([ATP}}_{\text{H}}{\text{]}}_{0}\;{\text{+ [ATP}}_{\text{C}}{\text{]}}_{0}\text{)}K_{e}}$$

Using equation 8 in the first of equation 2 and solving it with the initial condition that [HK~P_H_] = 0 at *t* = 0, we obtain

(9) $${\text{[HK\textasciitilde{}P}}_{\text{H}}\text{]}=\frac{{{\text{[ATP}}_{\text{H}}\text{]}}_{0}}{{{\text{[ATP}}_{\text{H}}\text{]}}_{0}{\text{+[ATP}}_{\text{C}}{\text{]}}_{0}}\phi {\text{[HK]}}_{0}\text{(1}-e^{-At}\text{)}$$

The evolution over time of the total radiolabeled HK species, [HK~P_H_] + [HK-ATP_H_], denoted [HK·P_H_], can then be written by combining equations 3 and 8 and adding equation 9:

(10) $$[HK\cdot P_{H}]=[HK\sim P_{H}]+[HK{\text{-ATP}}_{H}]=\frac{{\left[{\text{ATP}}_{H}\right]}_{0}}{{\left[{\text{ATP}}_{H}\right]}_{0}+{\left[{\text{ATP}}_{C}\right]}_{0}}\phi {\left[HK\right]}_{0}\left(1-\frac{e^{-At}}{1+({\left[{\text{ATP}}_{H}\right]}_{0}+{\left[{\text{ATP}}_{C}\right]}_{0})K_{e}}\right)$$

where we recall that $A=k_{p}K_{e}{\text{([ATP}}_{\text{H}}{\text{]}}_{0}{\text{+[ATP}}_{\text{C}}{\text{]}}_{0}\text{)}/(1+K_{e}{\text{([ATP}}_{\text{H}}{\text{]}}_{0}+{{\text{[ATP}}_{\text{C}}\text{]}}_{0}\text{))}$.

Equation 10 thus provides an analytical expression for the evolution of [HK·P_H_] over time when HK is limiting and [ATP_H_] and [ATP_C_] remain close to their initial values. When HK is not limiting, [ATP_H_] and [ATP_C_] cannot be assumed constant and the differential equations in equation 2 must be integrated along with the constraints in equations 3 and 4 to obtain the corresponding evolution over time. We next fit equation 10 to the mean values of the measured data of HK associated with labeled ATP using the Levenberg-Marquardt algorithm in MatLab with *k*_*p*_, *K*_*e*_, and ϕ as adjustable parameters. All the data for a given HK were fit simultaneously.

### Fits of mathematical model to data and determination of kinetic parameters.

**FIG 4:**
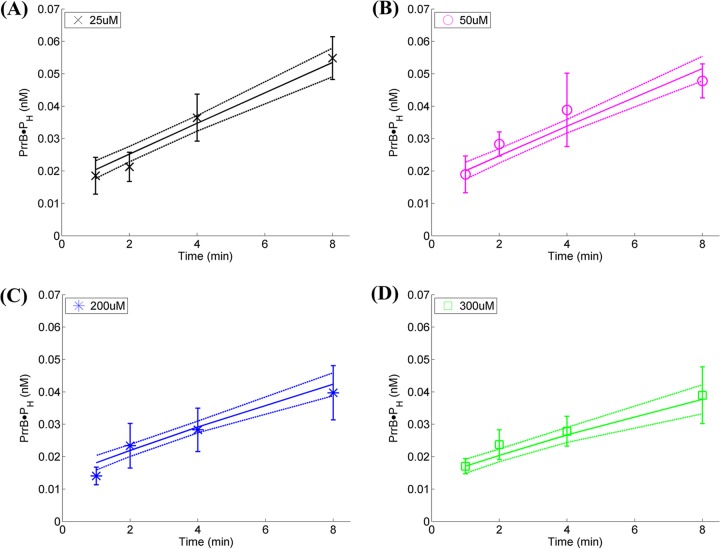
Fits of model predictions to data of PrrB autophosphorylation kinetics. Best-fits (solid lines) along with 95% confidence intervals (dashed lines) of model predictions (equation 10) to the data in Fig. 2 (symbols). Fits are obtained simultaneously to all the data obtained at various concentrations of unlabeled ATP as indicated in the panels. (A) 25 µM, (B) 50 µM, (C) 200 µM, and (D) 300 µM. (Overall *R*^2^ = 0.9.) The resulting parameter estimates are listed in Table 1. The data are shown as means ± SD (*n* = 3).

The model captured all the trends observed above and provided good fits to the experimentally obtained data of autophosphorylation of PrrB (Fig. 4), PhoR (Fig. S4), and MtrB (Fig. S5). The resulting best-fit parameter estimates along with their 95% confidence intervals are listed in Table 1. We note that for each HK, data of all the ATP levels were fit simultaneously. The same initial HK concentration was used for all the ATP levels in these fits, consistent with the experiments. Experimental uncertainties could introduce variations in the initial HK concentration. Indeed, where the agreement between the best-fit parameter estimates and the data appeared inadequate (such as for MtrB), allowing variation in the initial HK level restored the agreement (Fig. S6). We determined from the fits that of the three HKs studied, PhoR had the highest equilibrium association constant, *K*_*e*_ (2.7 mM^−1^), and hence associated with ATP the most strongly. PhoR was followed by MtrB (*K*_*e*_ = 1.7 mM^−1^) and PrrB (*K*_*e*_ = 0.5 mM^−1^) in the strength of this association. Following association with ATP, PrrB had the highest rate of autophosphorylation (*k*_*p*_ = 0.31 min^−1^) and was followed by MtrB (*k*_*p*_ = 0.27 min^−1^) and PhoR (*k*_*p*_ = 0.17 min^−1^). We also estimated the fraction ϕ of the HK proteins that was active and capable of association with ATP and autophosphorylation. We found ϕ to be the highest with MtrB (14%), followed by PrrB and PhoR, the lowest at ~3%. Note that the overall extent of autophosphorylation is determined by all of these parameter values, *viz.*, *K*_*e*_, *k*_*p*_ and ϕ, as described by our model (equation 10).

10.1128/mSphere.00111-18.4 FIG S4  Fits of model predictions to the data of PhoR autophosphorylation kinetics. Best-fits (solid lines) along with 95% confidence intervals (dashed lines) of model predictions (equation 10) to the data in Fig. 2 (symbols) is shown. Fits are obtained simultaneously to all the data obtained at various concentrations of nonradioactive ATP, *viz*., 10 µM (A), 15 µM (B), 100 µM (C), 150 µM (D), and 200 µM (E). The resulting parameter estimates are in Table 1. (Overall *R*^2^ = 0.8.) The data are shown as means ± SD (*n* = 3). Download FIG S4, TIF file, 1.3 MB. Copyright © 2018 Sankhe et al. 2018 Sankhe et al. This content is distributed under the terms of the Creative Commons Attribution 4.0 International license.

10.1128/mSphere.00111-18.5 FIG S5  Fits of model predictions to the data of MtrB autophosphorylation kinetics. Best-fits (solid lines) along with 95% confidence intervals (dashed lines) of model predictions (equation 10) to the data in Fig. 2 (symbols) are shown. Fits are obtained simultaneously to all the data obtained at various concentrations of nonradioactive ATP as indicated: 10 µM (A), 15 µM (B), 100 µM (C), 150 µM (D), and 200 µM (E). The resulting parameter estimates are listed in Table 1. (Overall *R*^2^ = 0.7.) The data are shown as means ± SD (*n* = 3). Download FIG S5, TIF file, 1.1 MB. Copyright © 2018 Sankhe et al. 2018 Sankhe et al. This content is distributed under the terms of the Creative Commons Attribution 4.0 International license.

10.1128/mSphere.00111-18.6 FIG S6  Model fitting to individual MtrB autophosphorylation data sets. The fits to data sets of MtrB autophosphorylation at 5 µM and 10 µM ATP concentration in Fig. 3 were redone with the initial HK concentration [HK]_0_ as an adjustable parameter. The other parameters were the same as in Table 1. The best-fits (solid lines) and 95% confidence intervals (dashed lines) are shown along with the data (symbols) shifted arbitrarily for clarity. The best-fit estimates of [HK]_0_ for 5 µM and 10 µM ATP data sets were 6.5 (6.2 to 6.8) µM and 3.5 (2.9 to 4.1) µM, respectively. Download FIG S6, TIF file, 0.4 MB. Copyright © 2018 Sankhe et al. 2018 Sankhe et al. This content is distributed under the terms of the Creative Commons Attribution 4.0 International license.

**TABLE 1:** Parameter estimates for autophosphorylation kinetics of various HKs of M. tuberculosis^a^

Histidine kinase	Parameter estimates^a^		
	*K*_*e*_ (mM^−1^)	*k*_*p*_ (min^−1^)	ϕ (%)
PrrB	0.5 (0.3–0.9)	0.31 (0.20–0.42)	5.2 (2.8–7.6)
PhoR	2.7 (1.2–4.2)	0.17 (0.06–0.28)	2.9 (1.6–4.2)
MtrB	1.7 (0.3–3.2)	0.27 (0.04–0.51)	14 (4.4–24)

a Best-fit parameter values (95% confidence intervals) obtained from fits of our model to data (Fig. 4 and Fig. S4 and S5 in the supplemental material).

### Validation of parameter estimates.

**FIG 5:**
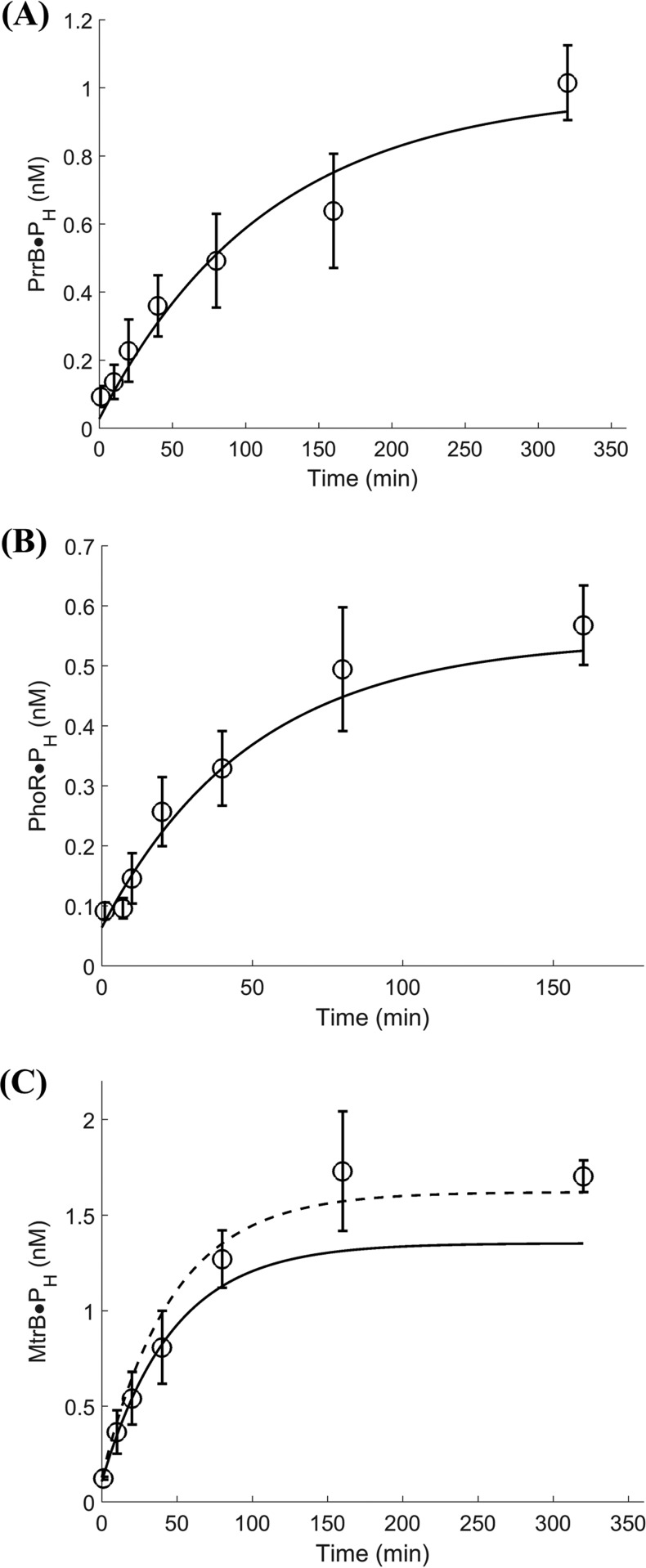
Validation of HK autophosphorylation kinetics with mathematical model. Model predictions (solid lines) using parameter values estimated in Fig. 3 (Table 1) compared with data (symbols) of labeled HK as indicated, (A) PrrB, (B) PhoR, and (C) MtrB from independent experiments using the HTA platform. Concentrations of HKs used for the reaction were 5 µM for MtrB and 10 µM for PrrB and PhoR along with 50 µM unlabeled ATP and ~95 nM labeled ATP. The data are shown as means ± SD (*n* = 3).

To validate the above parameter estimates, we performed independent experiments using the HTA platform, where the autophosphorylation reactions were performed for longer durations (up to 3 h for PhoR and up to 6 h for PrrB and MtrB) and using different initial HK and ATP concentrations. The reaction kinetics were then predicted using the best-fit parameter estimates above in equation 10. The resulting predictions were in close agreement with experimental data for all three HKs (Fig. 5). For instance, with PrrB, the measured [HK·P_H_] was ~1 ± 0.11 nM at 320 min, and the corresponding model prediction with the parameter values in Table 1 was 0.93 nM (Fig. 5A). Similarly, the measured PhoR·P_H_ was ~0.56 ± 0.06 nM at 160 min, while the corresponding model prediction using parameter values in Table 1 was 0.53 nM (Fig. 5B), and with MtrB, the measured [HK·P_H_] was ~1.7 ± 0.08 nM at 320 min, and the corresponding model prediction with the parameter values in Table 1 was 1.35 nM (Fig. 5C). The model underpredicts the data for MtrB but captures it using ϕ = 0.17 (dashed line) which is within the 95% confidence intervals on ϕ in Table 1.

**FIG 6:**
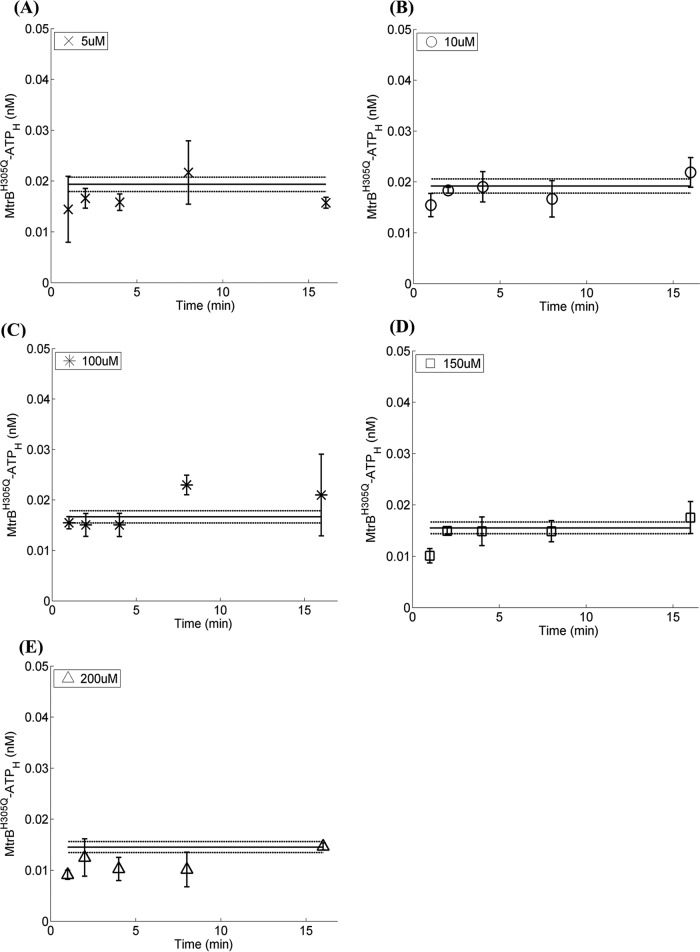
Validation of two-step HK autophosphorylation mechanism. Time course analysis of formation of labeled MtrB^H305Q^·ATP complex using the HTA platform in the presence of various concentrations of unlabeled ATP as shown in the panels. (A) 5 µM, (B) 10 µM, (C) 100 µM, (D) 150 µM, (E) 200 µM. A fixed concentration of labeled ATP (20 nM) and 5 µM mutated MtrB^H305Q^ was used for all reactions. Best-fits (solid lines) along with 95% confidence intervals (dashed lines) of model predictions (equation 10) are obtained by simultaneous fitting to the data using *k*_*p*_ = 0 (absence of phosphorylation), *K*_*e*_ = 1.7 mm^−1^, and ϕ as adjustable parameter. The data are shown as means ± SD (*n* = 3).

To further validate the two-step mechanism captured by the model, we performed independent experiments of the autophosphorylation kinetics with the phosphorylation-defective histidine mutant MtrB^H305Q^ which is proficient in ATP binding, with various concentrations of unlabeled ATP and a fixed concentration of labeled ATP ([ATP_H_] ~21 nM) using the HTA platform. In this experiment, it is anticipated that only the first step of the reaction, i.e., ATP binding, will be captured at all time points, and no contribution of the second step, i.e., phosphorylation, will be recorded and that is what was observed (Fig. 6). The data were fit using our model (equation 10) with *k*_*p*_ = 0, given the absence of phosphorylation, and with the best-fit parameter estimates of *K*_*e*_ above. ϕ was used as an adjustable parameter. The model provides good fits to the data at all concentrations of unlabeled ATP yielding ϕ = 9%, which is within the 95% confidence interval on ϕ of MtrB (Table 1). We also ruled out nonspecific ATP binding by using a mutant defective in ATP binding, MtrB^N419D^, which is also phosphorylation defective (Fig. S7). This close agreement between model predictions and independent experimental measurements provides a successful test of the two-step mechanism as well as of the parameters estimated using the HTA platform and our mathematical model.

10.1128/mSphere.00111-18.7 FIG S7  Validation of two-step autophosphorylation reaction and absence of nonspecific ATP binding. To negate the possibility of capturing nonspecific binding of ATP in the two-step model, we performed an independent phosphorylation kinetic analysis for MtrB and its phosphorylation-defective mutant, MtrB^H305Q^, and a mutant defective in ATP binding, MtrB^N419D^, with 100 µM nonradioactive ATP and ~30 nM radioactive ATP. (A) The equivalence of the retention of radiolabel alone on the membrane obtained in the negative control lacking the HK and the one obtained using the MtrB^N419D^ suggest the absence of any nonspecific binding. (B) The comparative analysis of the autophosphorylation kinetics observed in MtrB and MtrB^H305Q^ highlights the two-step activation mechanism of HK autophosphorylation. The data are shown as means ± SD (*n* = 3). Download FIG S7, TIF file, 0.2 MB. Copyright © 2018 Sankhe et al. 2018 Sankhe et al. This content is distributed under the terms of the Creative Commons Attribution 4.0 International license.

Overall, our findings reveal a two-step activation model for the bacterial HKs, which involves their association with ATP, an extremely rapid step, followed by a slower *cis* autophosphorylation reaction step.

## DISCUSSION

**FIG 7:**
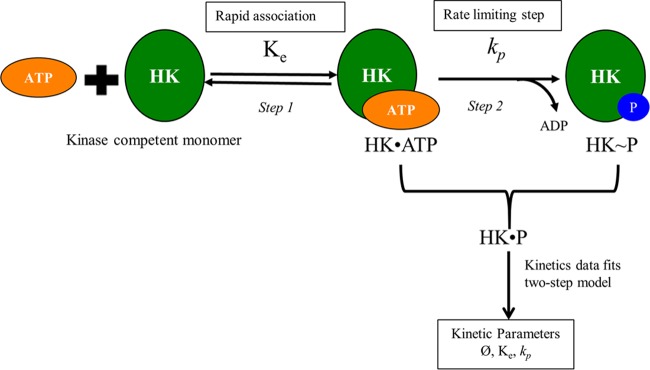
Summarized model of findings reported in this study. Step 1 indicates the presence of rapid and reversible association of ATP with HK. Step 2 is the slow autophosphorylation of HK in response to stimulation by ligand, which is the rate-limiting step. P, phosphate group.

On account of the prevailing view of the mechanism of autophosphorylation of HKs being the *trans* mode, rate measurements and modeling of the *cis* mode of phosphorylation have not been extensively explored. We present here experimental evidence to demonstrate the existence of the *cis* mode of autophosphorylation along with a combined theoretical and experimental analysis of the kinetics of autophosphorylation of three HKs of *M. tuberculosis* implicated in regulating its virulence. We found that *cis* autophosphorylation of the HKs was orchestrated via a two-step mechanism: rapid, reversible association of HK with ATP, followed by the phosphorylation of the HK by the associated ATP (Fig. 7), which has not been reported previously but at the same time was anticipated based on the structure-function properties of the HKs (15). We developed a mathematical model based on this mechanism that captured experimental data quantitatively. Experiments with mutant proteins provided a direct evidence of this two-step *cis* mechanism. Using fits of our model to the data, we estimated the rates of the autophosphorylation reaction, which allowed characterization of the HKs in terms of their relative propensity to be activated.

We employed a high-throughput assay (HTA) platform for our measurements, which yielded readouts in close agreement with classical PAGE/autoradiography but using ~10-fold-lower labeled ATP and ~20-fold-lower data processing time and where reactions can be run in parallel in 96 reactions per plate. Our model presents a single analytical expression, akin to the classical Michaelis-Menten formalism, that allows facile data analysis. A predictive rule for the mode of autophosphorylation (*cis* or *trans*) has been established structurally by studying the determinants of the loop handedness of the HKs (27). Our combined experimental and mathematical analysis may be readily scaled up to the large number of HKs that still remain poorly studied and may be autophosphorylated in *cis*.

Previous studies have reported *trans* autophosphorylation of several HKs (16, 18), in which dimerization precedes the reaction (10, 40). Although the autophosphorylation mechanism is conserved regardless of directionality as the same product, i.e., phosphorylated HK, which is necessary for the phosphorylation of a response regulator, the structural differences in the helix loops of the DHp domain dictating the directionality emphasize the necessity for a dimerization event for HK *trans* autophosphorylation (27). The bimolecular autocatalytic event involving ATP and HK as the substrates and phosphorylated HK within a dimer as the enzyme follows a random bi-bi mechanism which has been described using the classical Michaelis-Menten equations (21). Kinetics of HK autophosphorylation has been studied by employing such reaction schemes yielding kinetic parameters for ATP and HK as the substrate of HKs namely, CheA (11, 41, 42), NtrC (23), VanS (22), FixL (43), and other HKs from various organisms like Shewanella oneidensis (gene identifier [ID] or accession no. 1169886), and Legionella pneumophila (gene ID 19831845) (36). However, our experiments and many reported recently (19, 20, 25, 27, 44) indicate that some HKs undergo autophosphorylation by the *cis* mode, that the *cis* autophosphorylation is expected to be dimerization independent, and that the monomer can be considered a kinase-competent state of the HK (3) and can be modeled without requiring the dimerization process in the reaction steps. However, we recorded the presence of dimers even for HK, which undergoes autophosphorylation in *cis*, which was evident in the MST and other interaction experiments. The significance of this dimerization process is not clear at this time and does not conform with the models generated with *trans* autophosphorylation process. Further, the low fraction of HK that is active *in vitro* makes HK limiting even at the initial time points in our experiments and those reported previously (22–24). The Michaelis-Menten equations therefore could not be readily applied to such data. It is important to note here that the small active fraction of HKs that we obtained (ϕ of ~3 to 14%) is similar to those obtained for many other HKs, such as VanS of Enterococcus faecium (ϕ of ~10%) (22, 24) and the HK NtrC of Escherichia coli (ϕ of ~30%) (23), attributed to intrinsically low basal level of functional conformers, due to use of only the C-terminal domain of the HKs for activity assays. Dephosphorylation of HK~P may also underlie the low percentage activity of HK, but this appears negligible in our experiments given the close agreement between our predictions without adjustable parameters and experimental measurements of HK autophosphorylation over extended durations (Fig. 5). This consonance between our estimates of the active HK fraction and those reported previously, together with the validation of our model predictions using independent experiments (Fig. 5 and 6) gives us confidence in our approach and design of the rate equations.

Given that evidence of *cis* autophosphorylation of HKs is growing (19, 25, 44), our experimental workflow and mathematical analysis are expected to provide insights into the underlying reaction scheme and allow robust estimation of kinetic parameters of the rate. We expect that the kinetic parameters will provide inputs to theoreticians interested in analyzing the behavior of TCS systems and their implications for bacterial survival and adaptation. Simultaneously, the parameters would aid the comparative evaluation of drug targets and potentially accelerate antibacterial drug development.

## MATERIALS AND METHODS

### Materials.

Media, salts, and phenylmethylsulfonyl fluoride (PMSF) were purchased from SRL, India. Glycine, imidazole, and ATP were purchased from Merck Sigma-Aldrich, USA. Protein markers were purchased from Thermo Fisher Scientific, USA. Antibiotics, IPTG (isopropyl-β-d-1-thiogalactopyranoside) and DTT (dithiothreitol) were from GoldBio Inc., USA. The protease inhibitor cocktail was from Amresco, USA. Ni^2+^-NTA (nitrilotriacetic acid) (NTA) resin was from Qiagen, GmbH. The multiscreen filter plates (catalog no. MAHAN 4510) were from Millipore, USA, and γ-^32^P-labeled ATP (>3,000 Ci/mmol) was from BRIT-Jonaki, India.

### Bacterial strains.

For protein purification, E. coli Origami and Origami B (Novagen Inc., USA) were used. These strains carrying the recombinant plasmids were propagated in LB containing ampicillin (100 µg/ml). The recombinant plasmids used for protein overexpression have been reported previously (31). Briefly, for histidine kinases (HKs) MtrB, PrrB, and PhoR, the plasmids containing only the cytosolic catalytic domains were used. For GFP (green fluorescent protein)-tagged MtrB (MtrB-GFP), the GFP gene was cloned downstream of the cytosolic catalytic domain of MtrB (as described above) with a linker region which encoded the GSGGG spacer peptide, which facilitated functional separation of the two proteins.

### Site-directed mutagenesis.

Point mutations in the protein overexpression plasmids were introduced using the QuikChange site-directed mutagenesis protocol and Phusion polymerase (New England BioLabs, USA). The presence of mutations was confirmed by DNA sequencing.

### Recombinant protein purification.

For recombinant protein production, E. coli strains carrying the recombinant plasmid were grown at 37°C in 200 ml of 2× YT broth until an optical density at 600 nm (OD_600_) of 0.4 to 0.6 was reached, then IPTG (0.5 mM) was added to the culture, and the culture was incubated further for 15 to 20 h at 12 to 15°C for protein overexpression. Cells were harvested by centrifugation and stored until use at −80°C. For protein purification in soluble conditions, the protocol described previously was followed (31).

### Analysis of HK autophosphorylation mechanism (*cis* or *trans*) through PAGE/autoradiography.

The purified phosphorylation site mutant of HK protein MtrB, MtrB^H305Q^ (mutation in the DHp domain [35]) (5 µM), was coincubated with the mutant protein defective for ATP binding (mutation in the catalytic ATPase domain) of MtrB^N419D^ for 10 min in the autophosphorylation buffer (50 mM Tris-HCl [pH 8.0], 50 mM KCl, 10 mM MgCl_2_) at 30°C.The reaction was initiated by adding 50 µM ATP and 1 µCi of γ-^32^P-labeled ATP. The reaction was terminated after 60 min by adding 1× SDS-PAGE sample buffer (2% [wt/vol] SDS, 50 mM Tris-HCl [pH 6.8], 0.02% [wt/vol] bromophenol blue, 1% [vol/vol] β-mercaptoethanol, 10% [vol/vol] glycerol). The samples were resolved on 15% SDS-PAGE. After electrophoresis, the gel was washed and exposed to phosphor screen (Fujifilm Bas cassette2, Japan) for 4 h, followed by imaging with a Typhoon 9210 phosphorimager (GE Healthcare, USA).

### Determination of affinities of HK dimerization using MST.

Purified recombinant MtrB-GFP protein (50 nM) was mixed with increasing concentrations of titrant wild-type MtrB (0.76 nM to 25 µM) or mutant MtrB^H305Q^ (0.38 nM to 12.5 µM) or MtrB^N419D^ (0.38 nM to 6.25 µM) in the autophosphorylation buffer and kept at 30°C for 10 min. The sample was then loaded into standard treated capillaries and analyzed using a Monolith NT.115 instrument (NanoTemper Technologies GmbH). The blue laser was used for a duration of 35 s for excitation (microscale thermophoresis [MST] powerof 60%, light-emitting diode [LED] power of 40%). The data were analyzed using MO Control software (NanoTemper Technologies GmbH) to determine the dissociation constant (*K*_*D*_) for interacting proteins.

### HK dimerization analysis by nonreducing SDS-PAGE assay.

Recombinant GFP-tagged MtrB protein (5 µM) was coincubated with wild-type and mutant untagged MtrB proteins (2 µM) for 10 min in the autophosphorylation buffer at 30°C. The samples were treated with 2× nonreducing native sample buffer (Thermo Fisher Scientific, USA) and resolved by 15% SDS-PAGE. The protein bands were analyzed after Coomassie brilliant blue (CBB) staining and fluorescence imaging in epifluorescence model using epi-FITC illumination setting (ChemidocMP, Bio-Rad, USA). Since the proteins were subjected to nonreducing conditions, it may not be commensurate with the molecular markers used in reducing SDS-PAGE.

### CD spectroscopy.

Circular dichroism (CD) measurements were carried out on a Jasco spectropolarimeter. Residue molar ellipticity [θ] was defined as θ_obs_ (*lc*)^−1^, where θ_obs_ was the observed ellipticity, *l* was the light path length in centimeters, and *c* was the residue molar concentration of each protein. Briefly, all recombinant proteins at a concentration of 1 µM in 1× phosphate-buffered saline (PBS) buffer (137 mM NaCl, 2.7 mM KCl, 10 mM Na_2_HPO_4_, 1.8 mM KH_2_PO_4_) were subjected to CD analysis using a 0.1-cm cell.

### HK autophosphorylation using PAGE/autoradiography.

Purified HK (5 µM) was incubated in the autophosphorylation buffer containing 50 µM ATP and 1 µCi of γ-^32^P-labeled ATP at 30°C. The reaction was terminated at the desired time points (1 to 80 min) by adding 1× SDS-PAGE sample buffer. The samples were resolved on 15% (vol/vol) SDS-polyacrylamide gels. After electrophoresis, the gel was washed and exposed to phosphor screen (Fujifilm Bas cassette2, Japan) for 4 h, followed by imaging with a Typhoon 9210 phosphorimager (GE Healthcare, USA).

### HK autophosphorylation using the HTA platform.

Purified HK (5 to 10 µM) was incubated in autophosphorylation buffer (50 mM Tris Cl [pH 8.0], 50 mM KCl, 10 mM MgCl_2_) containing 50 µM ATP and 1 µCi of γ-^32^P-labeled ATP (>3,000 Ci/mmol; BRIT, India) at 30°C. The reaction was terminated at different time points (as indicated) by adding a modified stop buffer (1× stop buffer) (50 mM Tris-HCl [pH 8.0], 50 mM KCl, 10 mM EDTA, 0.1% [wt/vol] SDS) and stored on ice. The samples were transferred to a 96-well MultiScreen HTA plate prewetted with PBS (pH 7.4) and processed by high-throughput filtration using a vacuum manifold to remove the excess reaction components (mainly labeled ATP) and facilitate binding of the labeled proteins on the membrane. The wells were washed five times with 200 µl of cold PBS. The filters were air dried for 5 min, and individual filters were cut using the filter punching device (Millipore, USA) (45). The radioactivity retained on each filter was quantified by a liquid scintillation counter (PerkinElmer, USA) in the ^32^P channel using scintillation fluid (0.5% [wt/vol] 2,5-diphenyl oxazole and 0.005% [wt/vol] 1,4-bis-4-methyl-5-phenyl-2-benzene oxazoyl benzene in toluene). A negative-control reaction having the same components but lacking the HK was also processed on the high-throughput assay (HTA) platform in the same way to determine the retention of radiolabel alone on the membrane. At the same time, an internal control (same as the negative control) was directly used for scintillation counting to determine the exact disintegrations per minute (dpm) of the γ-^32^P-labeled ATP used per reaction. Experiments were also performed with different amounts of unlabeled ATP added initially (along with 1 µCi of γ-^32^P-labeled ATP) with fixed amounts of HK, and a time course experiment with fixed amounts of ATP and HK was also performed. Each experiment was performed in triplicate.

### Data fitting using mathematical model.

We fit equation 10 of our model (see below) to the mean values of the data of the concentrations of HK associated with labeled ATP measured as a function of time, following the start of the experiment. Data for various initial concentrations of unlabeled ATP for any given HK were fit simultaneously. *k*_*p*_, *K*_*e*_, and ϕ were used as adjustable parameters. The fitting was done using the Levenberg-Marquardt algorithm in MatLab using the inbuilt function NLINFIT. The fitting also yielded 95% confidence intervals on the parameters as well as the best-fit predictions. The goodness of fit was estimated by computing *R*^2^ values. The fit parameters were also tested by comparing model predictions with independent experiments with different initial concentrations of the reactants and run for extended durations.
