# Supplementary figures and images for: Activation of Bacterial Histidine Kinases: Insights into the Kinetics of the cis Autophosphorylation Mechanism
Source: mSphere. 2018 May 16;3(3):e00111-18. doi: 10.1128/mSphere.00111-18 (PMC5956149; doi:10.1128/mSphere.00111-18)

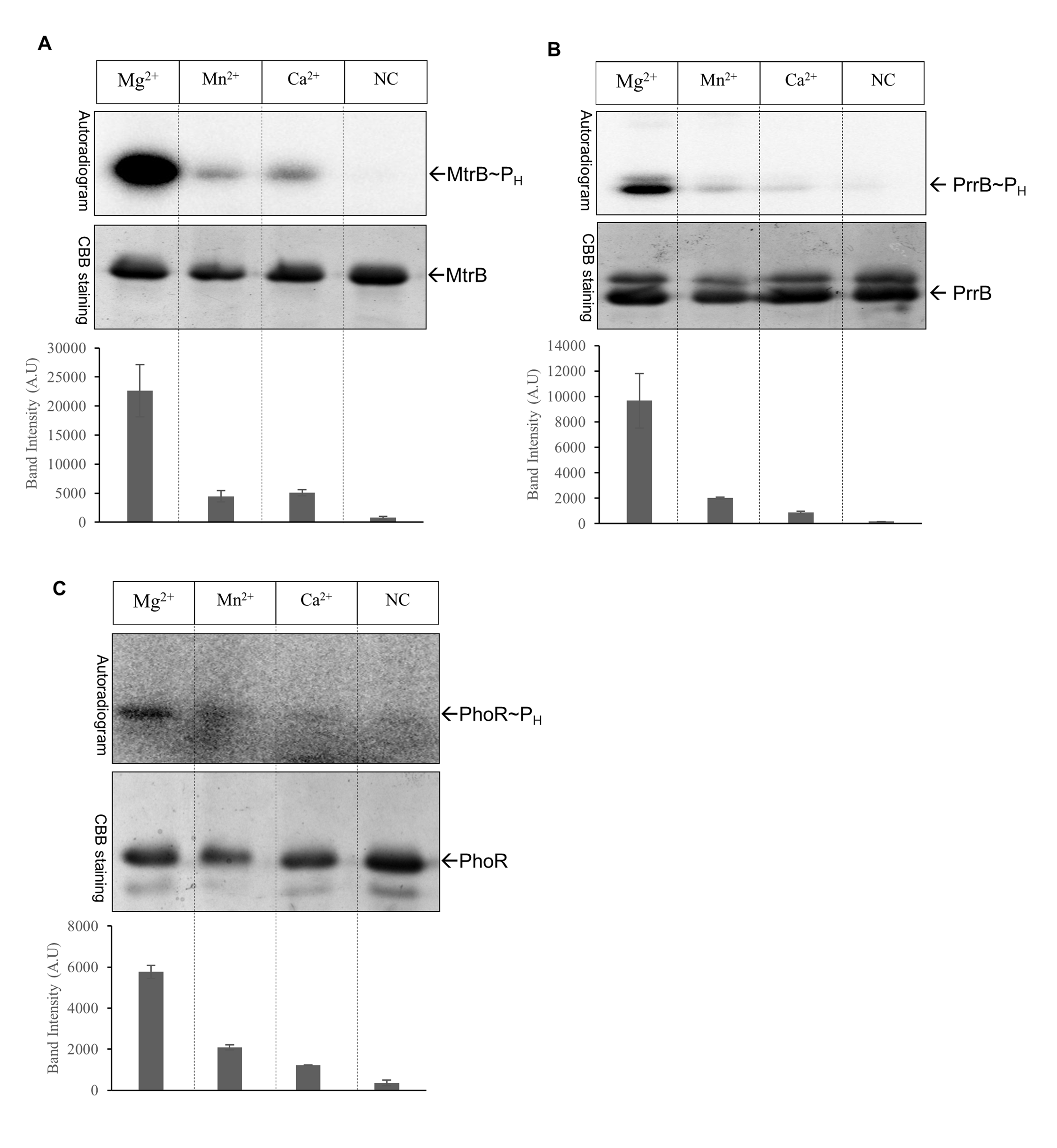

Supplement: FIG S1 [file sph003182544sf1.tif]

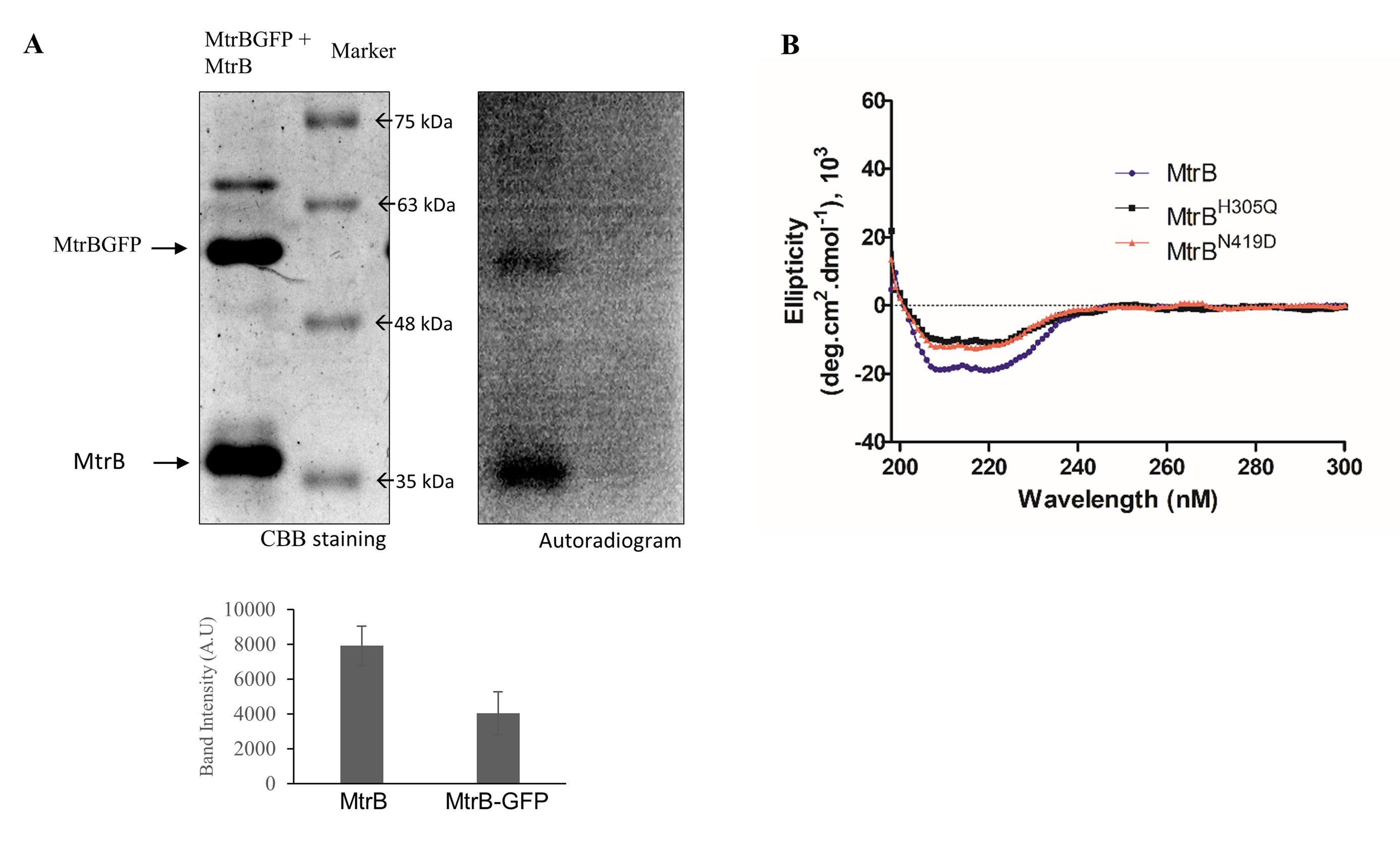

Supplement: FIG S2 [file sph003182544sf2.tif]

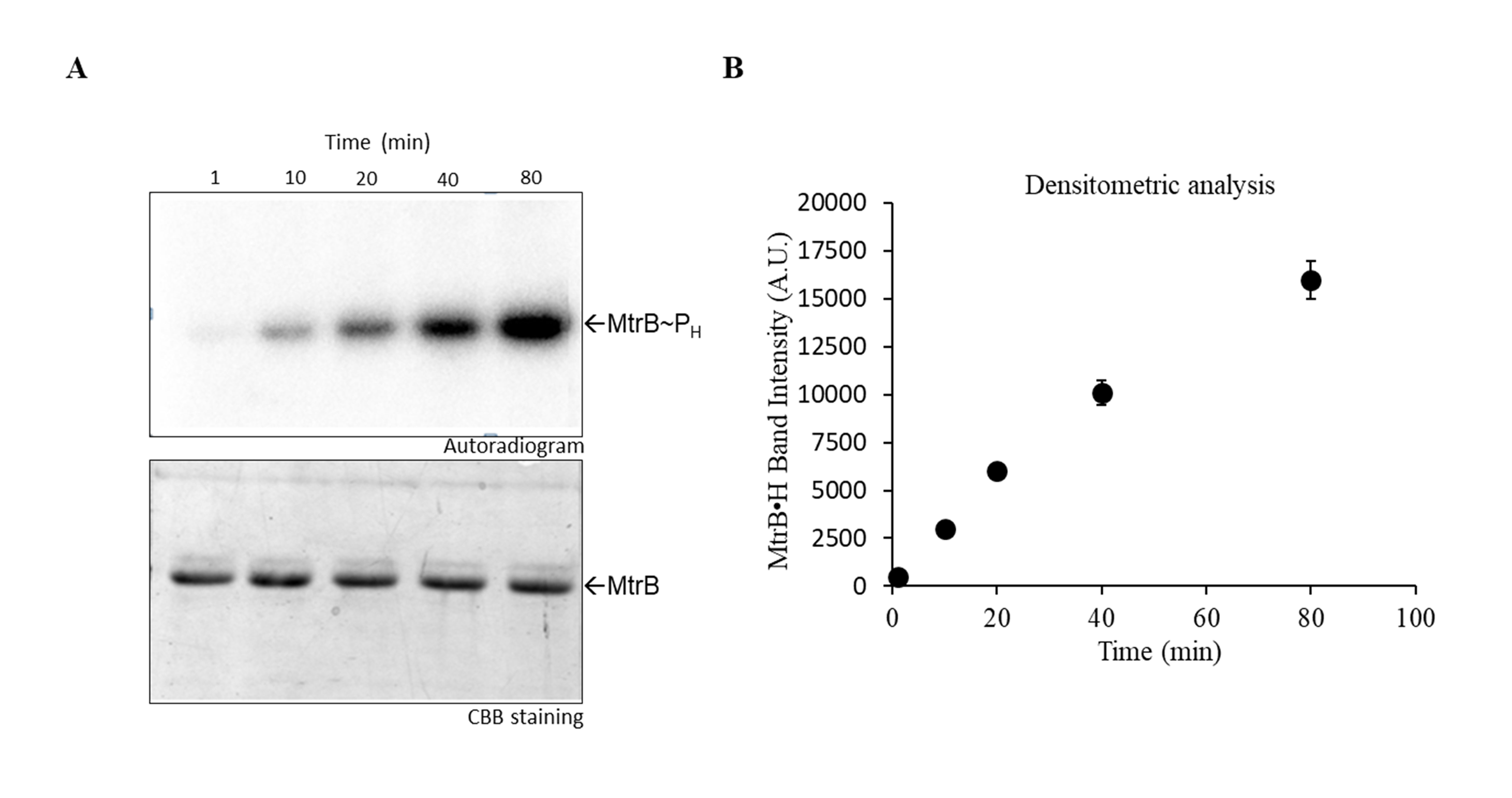

Supplement: FIG S3 [file sph003182544sf3.tif]

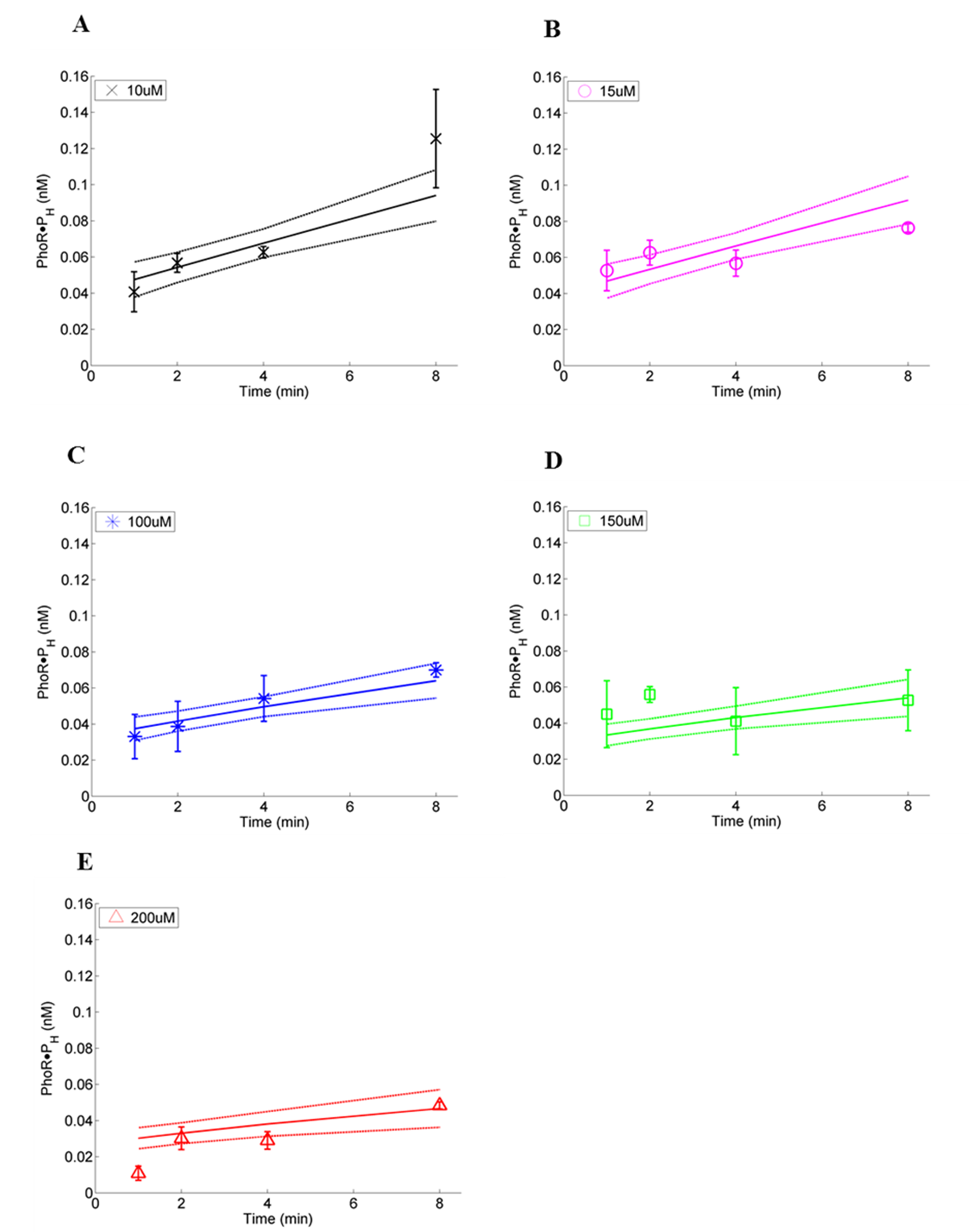

Supplement: FIG S4 [file sph003182544sf4.tif]

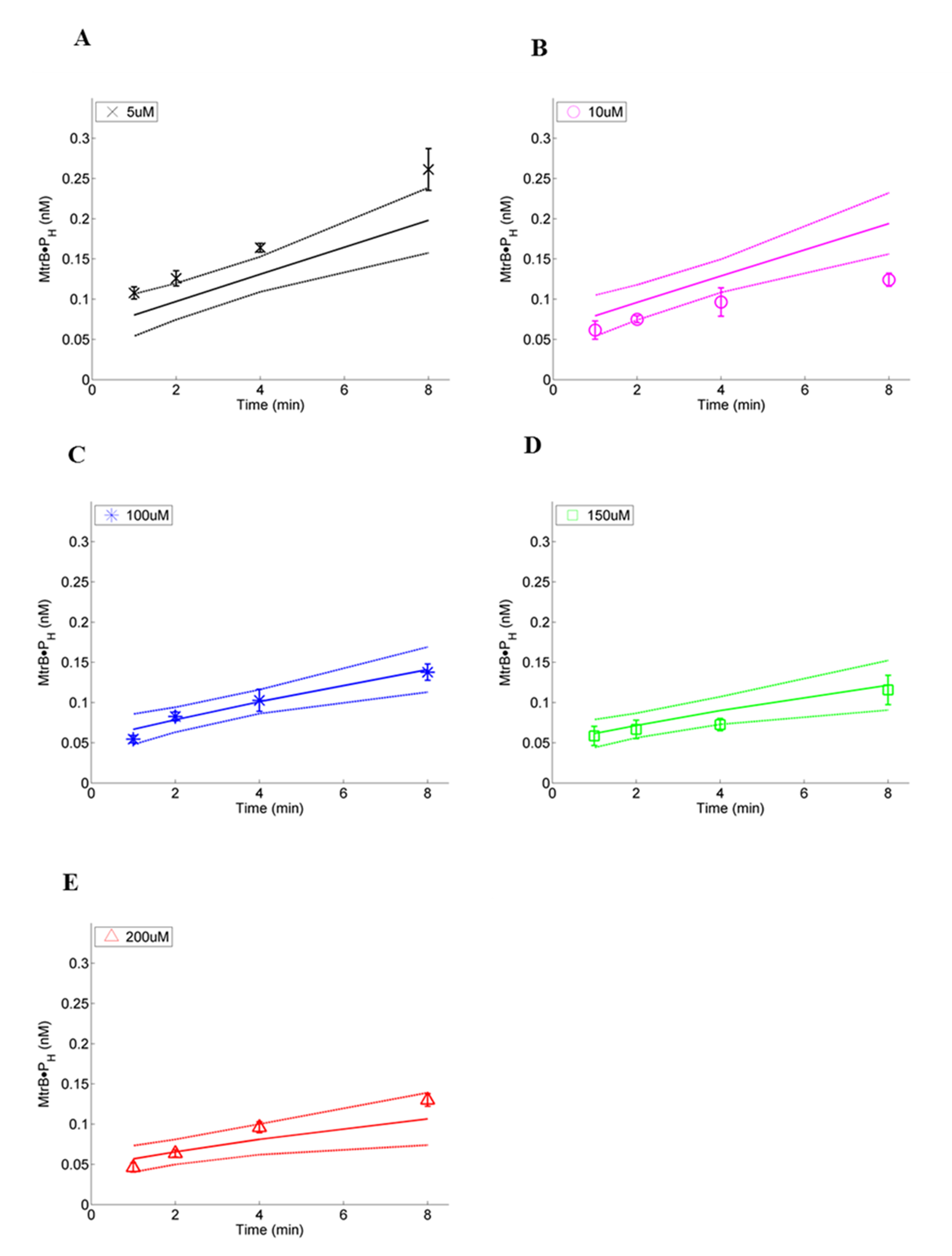

Supplement: FIG S5 [file sph003182544sf5.tif]

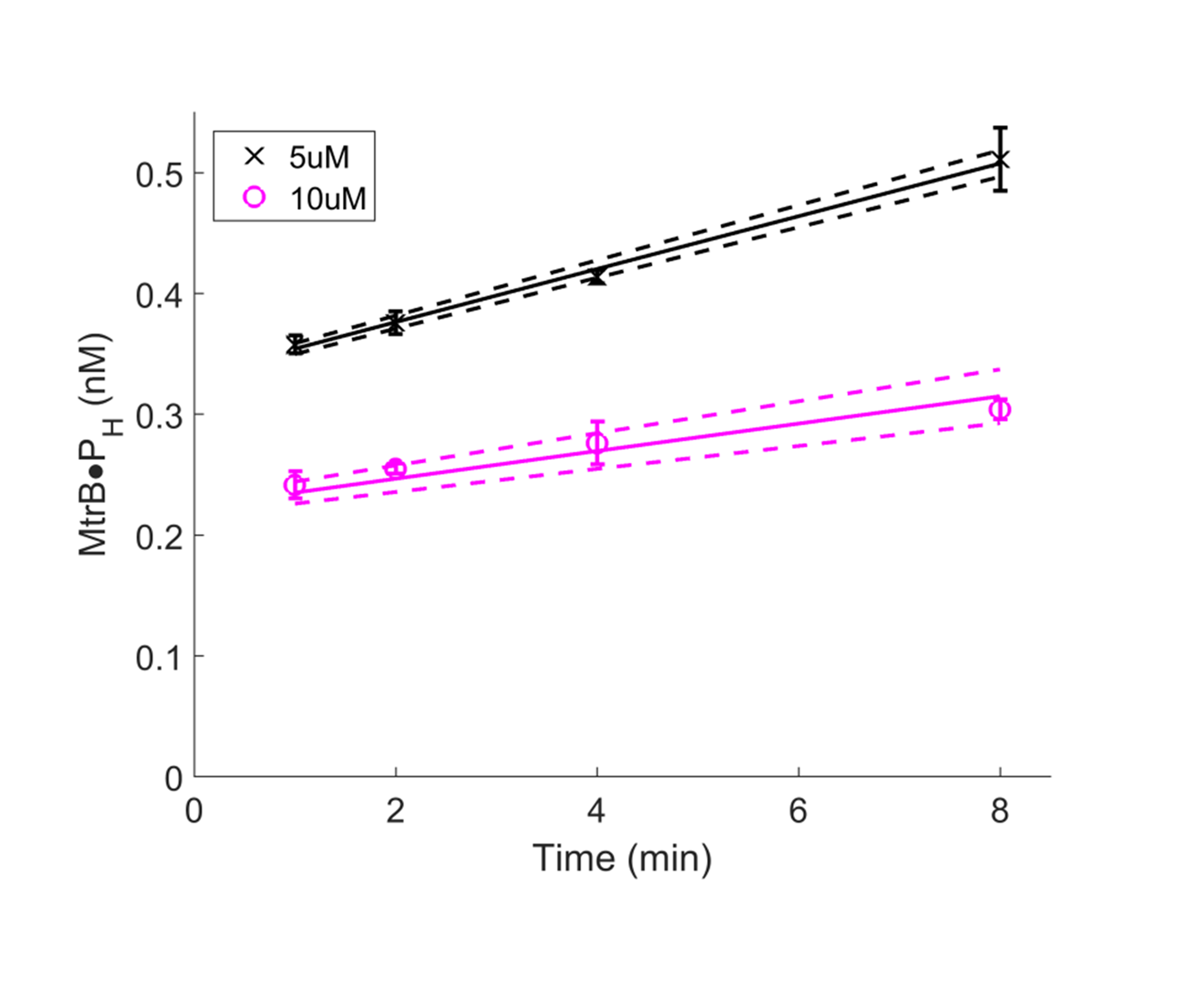

Supplement: FIG S6 [file sph003182544sf6.tif]

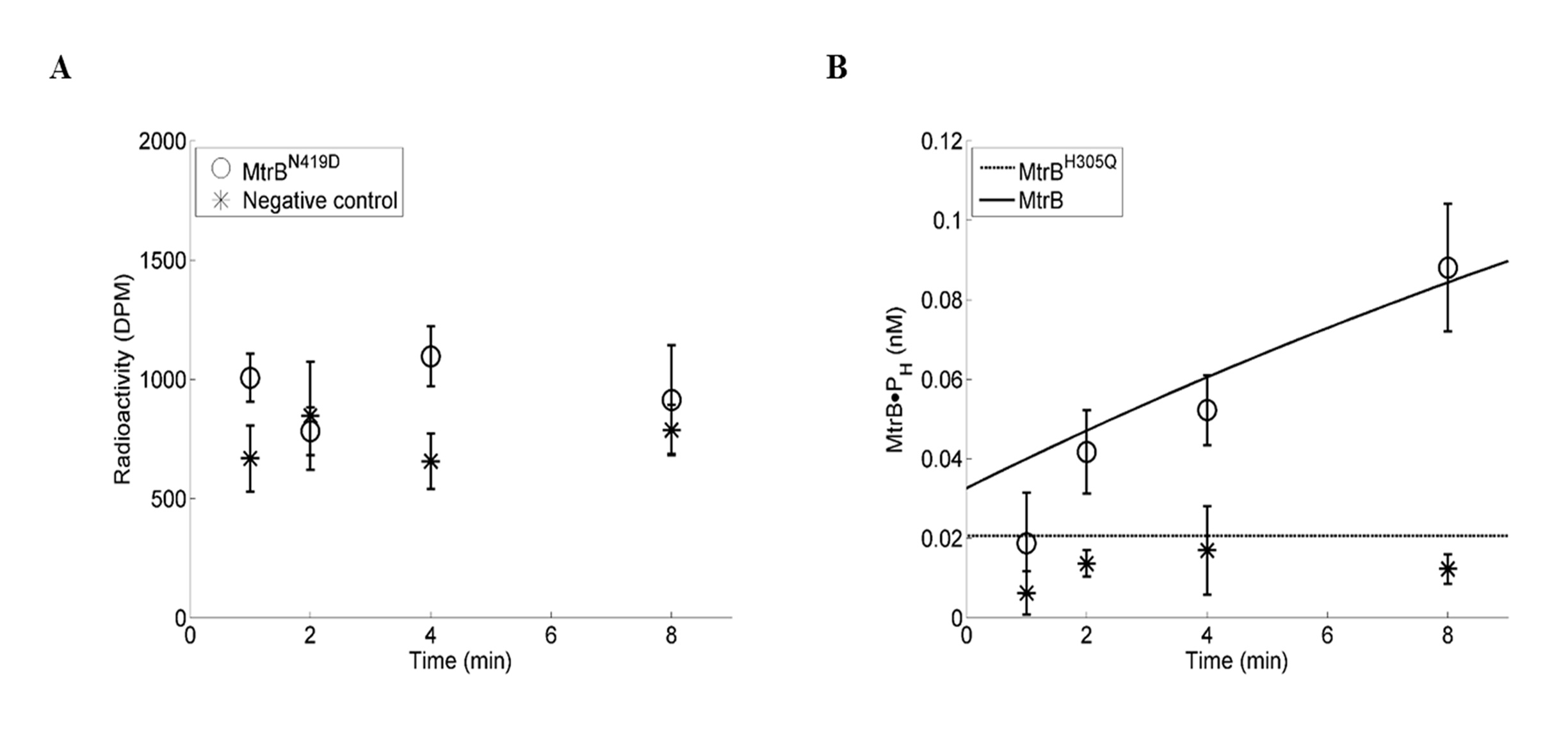

Supplement: FIG S7 [file sph003182544sf7.tif]
